# Symbiotic microbes affect the expression of male reproductive genes in *Glossina m. morsitans*

**DOI:** 10.1186/s12866-018-1289-2

**Published:** 2018-11-23

**Authors:** Francesca Scolari, Geoffrey Michael Attardo, Emre Aksoy, Brian Weiss, Grazia Savini, Peter Takac, Adly Abd-Alla, Andrew Gordon Parker, Serap Aksoy, Anna Rodolfa Malacrida

**Affiliations:** 1 Department of Biology and Biotechnology, University of Pavia, 27100 Pavia, Italy; 2 Yale School of Public Health, Department of Epidemiology of Microbial Diseases, New Haven, CT 06520 USA; 3 Present Address: Department of Entomology and Nematology, University of California Davis, Davis, CA 95616 USA; 4 Section of Molecular and Applied Zoology, Institute of Zoology, Slovak Academy of Sciences, 845 06 Bratislava, SR Slovakia; 5 International Atomic Energy Agency, Joint FAO/IAEA Division of Nuclear Techniques in Food and Agriculture, IPC Laboratory, A-1400 Vienna, Austria

**Keywords:** Tsetse, Endosymbionts, Aposymbiotic, Testes, MAGs, Ejaculate, Spermatophore

## Abstract

**Background:**

Tsetse flies (Diptera, Glossinidae) display unique reproductive biology traits. Females reproduce through adenotrophic viviparity, nourishing the growing larva into their modified uterus until parturition. Males transfer their sperm and seminal fluid, produced by both testes and male accessory glands, in a spermatophore capsule transiently formed within the female reproductive tract upon mating. Both sexes are obligate blood feeders and have evolved tight relationships with endosymbionts, already shown to provide essential nutrients lacking in their diet. However, the partnership between tsetse and its symbionts has so far been investigated, at the molecular, genomic and metabolomics level, only in females, whereas the roles of microbiota in male reproduction are still unexplored.

**Results:**

Here we begin unravelling the impact of microbiota on *Glossina m. morsitans* (*G. morsitans*) male reproductive biology by generating transcriptomes from the reproductive tissues of males deprived of their endosymbionts (aposymbiotic) via maternal antibiotic treatment and dietary supplementation. We then compared the transcriptional profiles of genes expressed in the male reproductive tract of normal and these aposymbiotic flies. We showed that microbiota removal impacts several male reproductive genes by depressing the activity of genes in the male accessory glands (MAGs), including sequences encoding seminal fluid proteins, and increasing expression of genes in the testes. In the MAGs, in particular, the expression of genes related to mating, immunity and seminal fluid components’ synthesis is reduced. In the testes, the absence of symbionts activates genes involved in the metabolic apparatus at the basis of male reproduction, including sperm production, motility and function.

**Conclusions:**

Our findings mirrored the complementary roles male accessory glands and testes play in supporting male reproduction and open new avenues for disentangling the interplay between male insects and endosymbionts. From an applied perspective, unravelling the metabolic and functional relationships between tsetse symbionts and male reproductive physiology will provide fundamental information useful to understanding the biology underlying improved male reproductive success in tsetse. This information is of particular importance in the context of tsetse population control via Sterile Insect Technique (SIT) and its impact on trypanosomiasis transmission.

**Electronic supplementary material:**

The online version of this article (10.1186/s12866-018-1289-2) contains supplementary material, which is available to authorized users.

## Background

Among Diptera, tsetse flies (*Glossina* spp.) display unique reproductive biology traits, including adenotrophic viviparity [1] and ejaculate transfer through a spermatophore capsule transiently formed within the female uterus [2, 3]. In addition, both sexes in tsetse are obligate blood feeders, and, as such, have evolved relationships with obligate symbionts providing essential nutrients lacking in their vertebrate blood diet [4–6]. These symbionts influence multiple aspects of tsetse female physiology, including nutrition, fecundity and immunity. To date, three main endosymbionts have been described in tsetse with one, *Wigglesworthia glossinidia*, being an obligate association found in all tsetse examined. *Wigglesworthia glossinidia* provides key metabolites, including vitamins, known to be present at low titers in the vertebrate blood [4]. Absence of *Wigglesworthia* results in larval abortion due in part to depletion of components of the B vitamin complex (specifically vitamins B1, B2 and B6), necessary co-factors for key metabolic pathways in *G. morsitans* [7]. Additional genetic/physiological adaptations include reliance on chaperonins for protein synthesis in *Wigglesworthia,* and enrichment of nutrient transport mechanisms by *Glossina*’s bacteriocytes. These findings further support the direct and indirect obligate interdependencies between tsetse and *Wigglesworthia*. This long-term co-evolutionary association has led to a multitude of dynamic molecular and biochemical interactions that ensures the optimal fitness of the partnership [4]. It remains to be determined whether the absence of *Wigglesworthia* has adverse nutritional effects on male biology that impact reproductive success. The commensal endosymbiont *Sodalis glossinidius* is widely distributed in various tissues, including the testes. While both *Sodalis* and *Wigglesworthia* undergo maternal transmission to the developing larva via the milk secretions of the mother [8], *Sodalis* can also be paternally transmitted through the ejaculate [9]. However, the role of *Sodalis* in tsetse’s male reproductive physiology remains unknown. This aspect is of particular interest in tsetse as recent works begin to unravel the role of facultative endosymbionts on male reproductive fitness in other insects [10]. As occurs in many other insect taxa, the gonads of the tsetse species *G. morsitans* also harbour the transovarially-transmitted *Wolbachia pipientis*, which can manipulate the reproductive biology of their hosts through multiple mechanisms, including cytoplasmic incompatibility (CI), which was noted in tsetse [11, 12]. *Wolbachia* prevalence in wild *G. morsitans* populations ranges from 9.5 to 100% [13] and such infection dynamics could be a result of the extensive genetic structuring estimated for tsetse populations [14, 15]. Moreover, three *Wolbachia* chromosome insertions have been identified in *G. m. morsitans* nuclear genome, with the two largest fragments carrying several putatively functional coding sequences [16]. These include genes encoding proteins with ankyrin repeat domains, considered to play a relevant role in *Wolbachia*-host interactions, despite their involvement in CI has not been confirmed yet [17–19]. Although the molecular basis of CI remains unknown, in *Drosophila*, *Wolbachia* has been shown to affect gene transcription in larval testes [20] and impact seminal fluid protein expression [21]. *Wolbachia* also affect the expression of immunity genes in a parasitoid wasp [22] and in mosquitoes [23]. It accomplishes this by influencing levels of host microRNAs (miRNA) that target specific host proteins as well as producing small RNAs that regulate mosquito gene expression [24, 25]. However, the potential effects of this bacterium on ejaculate composition and function, and thus on male reproductive success, remain unknown. This knowledge gap in tsetse is exacerbated by the fact that the symbiotic dialogue supporting the fitness of the partnership between tsetse and its symbionts has so far been investigated, at the molecular, genomic and metabolomics level, only in females.

Given the increasingly growing data supporting the role of the microbiota in the modification of host mating signals directly affecting mate choice (see [26] for a review), and our recent data showing the impact of *Wigglesworthia* on carbohydrate and amino acid metabolism [4], we hypothesize the presence of a direct effect of microbiota on *G. morsitans* male reproductive biology. This idea is further supported by data obtained from other insect species, which suggest that male macronutrient intake, carbohydrates in particular, influences sperm number [27]. To test this hypothesis, here we have taken advantage of the ability to generate fertile tsetse laboratory lines lacking endosymbionts (aposymbiotic), developed through the treatment of female flies with antibiotics and dietary yeast extract supplementation, which rescues *Wigglesworthia*-induced sterility [12, 28]. These flies produce offspring lacking all symbiotic bacteria. We compared the transcriptional profiles of genes expressed in the male reproductive tract of normal and aposymbiotic flies. Our previous transcriptomic and proteomic analyses revealed the protein composition of the spermatophore in *G. morsitans* and determined the tissues of origin of these proteins within the reproductive tract (testes and/or male accessory glands (MAGs)) of males with full microbiota [3]. This work suggested that these two reproductive tissues both contribute to the formation of spermatophore components, with MAGs producing highly abundant proteins of unknown function and the testes contributing a more diverse array of less abundant proteins. Considering the tight metabolic relationships established between tsetse and its endosymbionts, it is likely that this obligate symbiosis affects host physiological pathways important for male reproductive functions. Aposymbiotic males remain fertile and produce progeny [12], however the molecular impact of male aposymbiosis is unknown. Here we show that the absence of microbiota impacts a number of male reproductive genes by depressing gene activity in the MAGs, including sequences encoding seminal fluid proteins, and by increasing genes expression in the testes. Interestingly, our findings mirror the complementary roles MAGs and testes play in supporting male reproduction and open new avenues for disentangling the interplay between male insects and endosymbionts.

## Results

### Symbiont presence affects the expression of male reproductive genes

**Fig. 1:**
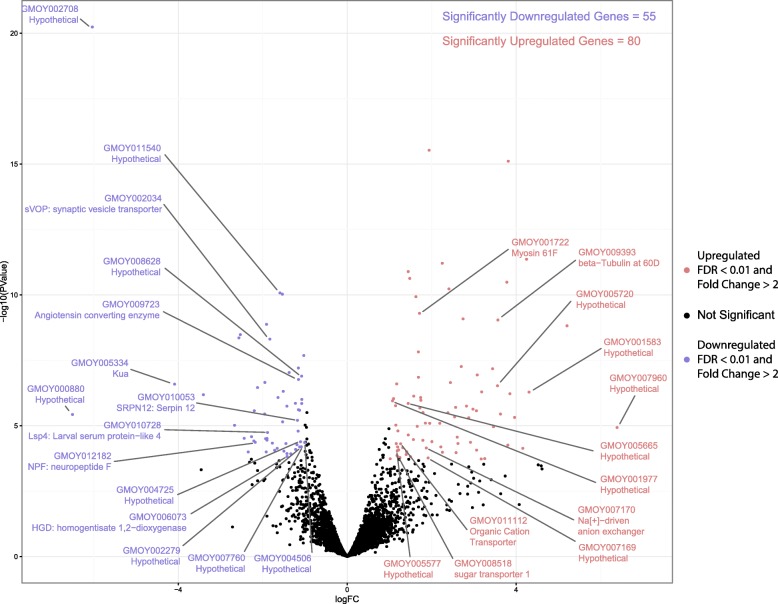
Volcano plot of differentially expressed genes in male reproductive tract tissue between wild-type and aposymbiotic *Glossina* males. This plot displays the fold change (log2) and p-value (log10) of differential transcript abundance of genes expressed in male reproductive tissues. Each point represents the change in a single transcript under aposymbiotic conditions. Blue points represent down-regulated genes and red points represent up-regulated genes with a fold change equal or greater than 2 and a false discovery rate score of less than or equal to 0.01. Black points are transcripts falling outside of the cut-off values. Labelled genes represent genes of interest discussed in this manuscript

**Fig. 2:**
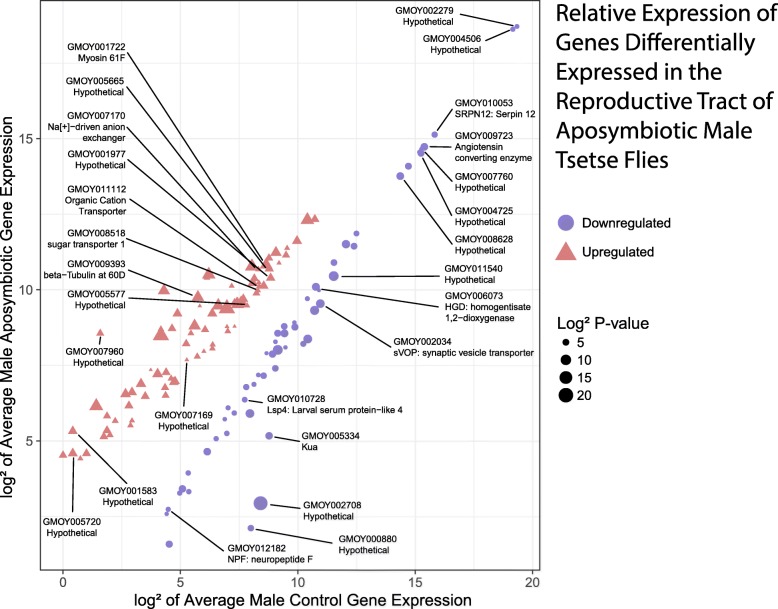
Scatter plot illustrating the relative expression levels of male reproductive genes differentially expressed under aposymbiotic conditions. The x-axis represents the log2 of the counts per million (CPM) expression value for wild-type flies. The y-axis represents the log2 value of CPM value for aposymbiotic flies. Red triangles and blue circles represent genes up and down-regulated respectively under aposymbiotic conditions. Point size represents the log2 of the *P*-value score for differential expression. Labelled points represent genes discussed in this manuscript

To determine whether endosymbionts play a role on the expression of male reproductive genes in *G. morsitans*, aposymbiotic (Gmm^Apo^) individuals were generated as progeny of tetracycline-treated females. Their symbiont-free status was verified by PCR assays for the presence of the three endosymbionts of tsetse, i.e. *Wigglesworthia*, *Sodalis* and *Wolbachia*, using bacterium-specific primers [12]. The PCRs clearly showed the absence of all three endosymbionts from both male and female flies (Additional file 1). These Gmm^Apo^ males, as well as males with a full symbiont complement (Gmm^WT^, control), were then used for the generation of transcriptomes from reproductive tissues (including the accessory glands, testes and ejaculatory duct), in three biological replicates [29]. About 19 million high-quality reads were obtained from each replicate. Reads were mapped to the predicted transcripts dataset from the *G. morsitans* genome as previously described [3]. A total of 8353 transcripts were identified as being significantly expressed in the reproductive tract. Principal Component Analysis (PCA) performed to compare global gene expression between the libraries demonstrated that the expression was structured according to the presence/absence of symbionts (Additional file 2). Indeed, the Gmm^Apo^ and the Gmm^WT^ libraries separated along the first principal component, which explained 59% of the variance. The transcripts were then categorized as significantly up- or down-regulated in Gmm^Apo^ versus Gmm^WT^ reproductive tissues by edgeR analysis (*P* < 0.05 and false discovery rate (FDR) score < 0.01). A total of 135 genes were identified as differentially expressed. Of those, 55 were down-regulated and 80 were up-regulated in the Gmm^Apo^ male tissues (Fig. 1, Additional file 3). Both up- and down-regulated gene datasets displayed similar distributions across the functional classes of the Gene Ontology (GO) categories (Additional files 4, 5 and 6). In the Biological Process GO, the most represented classes were cellular, metabolic and single-organism processes, while in the Molecular Function catalytic activity and binding were the predominant GO classes. Interestingly, about 50% of the up-regulated genes could be related to the membrane term (Cellular Component). It is noteworthy that a different trend of transcriptional variation, in relation to the controls, has been observed between up- and down-regulated genes. Indeed, as shown in Fig. 2, up-regulated genes tended to cluster within a relatively narrow expression variation interval (log2 ranging from about 5 to 12), whereas the variation interval of down-regulated genes was broader with some very highly expressed genes being represented (log2 ranging from about 4 to 20).

The availability of libraries previously generated from Gmm^WT^ MAG and testes allowed us to assess the preferential transcription of the genes that were up- and down-regulated in Gmm^Apo^ males in these two male tissues [3] (Additional file 3). Out of the 55 down-regulated genes, 22 (40%) displayed a biased expression in the MAGs (Table 1), while only five were associated with the testes (Table 2). The remaining 28 genes (51%) displayed an unbiased transcriptional profile between these two tissues. Conversely, out of 80 up-regulated genes, 68 (85%) displayed a biased expression in the testes (Table 3), and only one (i.e. GMOY011112) in the MAGs. The remaining 11 genes (14%) displayed an unbiased transcriptional profile between these two tissues.

**Table 1:** Twenty-two down-regulated genes in Gmm^Apo^ males showing a MAG-biased profile in Gmm^WT^ males

Vectorbase gene ID	Vectorbase Description	Best *nr* BLASTx Hit	Accession	BLASTx E-Value	MAGs/Testes fold change
GMOY000880		hypothetical protein FF38_03138, partial [*Lucilia cuprina*]	KNC27735.1	7.00E-79	14.90
GMOY002216		PREDICTED: uncharacterized protein LOC101892654 isoform X4 [*Musca domestica*]	XP_019890273.1	0.0	7.26
GMOY002279		PREDICTED: male accessory gland serine protease inhibitor [*Musca domestica*]	XP_005182681.1	5.00E-11	72.55
GMOY002397		hypothetical protein FF38_12465 [*Lucilia cuprina*]	KNC32328.1	5.00E-15	35.33
GMOY002708		PREDICTED: uncharacterized protein LOC101890794 isoform X4 [*Musca domestica*]	XP_005190518.1	1.00E-14	44.56
GMOY004506		no hits			117.35
GMOY004725		PREDICTED: male accessory gland serine protease inhibitor-like [*Stomoxys calcitrans*]	XP_013110809.1	7.00E-19	133.03
GMOY005334	Kua	PREDICTED: transmembrane protein 189 [*Stomoxys calcitrans*]	XP_013101683.1	3.00E-167	28.86
GMOY005914		hypothetical protein FF38_03123, partial [*Lucilia cuprina*]	KNC27757.1	0.0	5.32
GMOY006073	HGD: homogentisate 1,2-dioxygenase	homogentisate 1,2-dioxygenase [*Lucilia cuprina*]	KNC25382.1	0.0	14.27
GMOY006405	Ecdysone-inducible gene E3	PREDICTED: uncharacterized protein LOC106621464 [*Bactrocera oleae*]	XP_014095828.1	6.00E-88	26.08
GMOY006513	Zinc/iron regulated transporter-related protein 42C.2	putative zinc transporter 4 ic-like protein [*Haematobia irritans*]	JAV17535.1	6.00E-138	16.99
GMOY007760		no hits			95.61
GMOY008628		hypothetical protein FF38_13303 [*Lucilia cuprina*]	KNC29468.1	6.00E-16	56.42
GMOY009723	Angiotensin converting enzyme	angiotensin-converting enzyme [*Lucilia cuprina*]	KNC21161.1	0.0	33.37
GMOY010053	SRPN12: serine protease inhibitor (serpin) 12	PREDICTED: antichymotrypsin-2-like isoform X14 [*Stomoxys calcitrans*]	XP_013104701.1	4.00E-56	84.59
GMOY010303		PREDICTED: serine protease inhibitor Kazal-type 1 [*Musca domestica*]	XP_005190386.1	3.00E-17	5.77
GMOY010506	Putative thioesterase	PREDICTED: protein THEM6 [*Musca domestica*]	XP_005178985.1	3.00E-100	21.19
GMOY010906	kokopelli	hypothetical protein FF38_08330, partial [*Lucilia cuprina*]	KNC25973.1	4.00E-118	45.56
GMOY011540		putative uridine phosphorylase [*Haematobia irritans*]	JAV16233.1	6.00E-137	17.48
GMOY012067	Putative salivary secreted peptide	no hits			258.22
GMOY012281		odorant binding protein [*Calliphora stygia*]	AID61309.1	9.00E-04	20.09

**Table 2:** Five down-regulated genes in Gmm^Apo^ males showing a testes-biased profile in Gmm^WT^ males

Vectorbase gene ID	Vectorbase Description	Best *nr* BLASTx Hit	Accession	BLAST_E-Value	Testes/MAGs fold change
GMOY002034	synaptic vesicle transporter	hypothetical protein FF38_11887 [*Lucilia cuprina*]	KNC24952.1	0.0	613.26
GMOY008588		no hits			5.66
GMOY011064		hypothetical protein FF38_07287 [*Lucilia cuprina*]	KNC32510.1	0.0	6.06
GMOY011412	Aldehyde oxidase	PREDICTED: indole-3-acetaldehyde oxidase [*Musca domestica*]	XP_019893881.1	0.0	5.59
GMOY012182	NPF: neuropeptide F	hypothetical protein FF38_11880 [*Lucilia cuprina*]	KNC24956.1	7,00E-23	193.57

**Table 3:** Sixty-eight up-regulated genes in Gmm^Apo^ males showing a testes-biased profile in Gmm^WT^ males

Vectorbase gene ID	Vectorbase Description	Best *nr* BLASTx Hit	Accession	BLAST_E-Value	Testes/MAGs fold change
GMOY000619	Tetraspanin	hypothetical protein FF38_09930 [*Lucilia cuprina*]	KNC31071.1	3.00E-99	48.83
GMOY000812		PREDICTED: glucose dehydrogenase [FAD, quinone] [*Musca domestica*]	XP_005186034.1	0.0	72.42
GMOY001391	SCARA5: Scavenger Receptor Class A, Member 5	hypothetical protein FF38_05500 [*Lucilia cuprina*]	KNC27261.1	0.0	33.83
GMOY001583		integrin alpha-PS3 [*Lucilia cuprina*]	KNC31990.1	4.00E-17	135.91
GMOY001722	Myosin 61F	myosin-IB [*Lucilia cuprina*]	KNC31972.1	0.0	40.45
GMOY001794	fused lobes	putative beta-hexosaminidase fdl [*Lucilia cuprina*]	KNC26699.1	0.0	10.42
GMOY001977		hypothetical protein FF38_05070 [*Lucilia cuprina*]	KNC29694.1	0.0	23.94
GMOY002057		hypothetical protein FF38_02107 [*Lucilia cuprina*]	KNC28398.1	0.0	6.40
GMOY002247	nord	PREDICTED: uncharacterized protein LOC101887233 [*Musca domestica*]	XP_005185286.1	0.0	14.60
GMOY002466		peroxidase [*Lucilia cuprina*]	KNC34773.1	0.0	25.87
GMOY002491		protein takeout [*Lucilia cuprina*]	KNC23528.1	2.00E-67	44.31
GMOY002881		hypothetical protein FF38_01910 [*Lucilia cuprina*]	KNC34784.1	1.00E-174	223.02
GMOY002886		no hits			13.05
GMOY002911		hypothetical protein FF38_03087 [*Lucilia cuprina*]	KNC32537.1	8.00E-67	7.46
GMOY003353		PREDICTED: arylsulfatase B [*Musca domestica*]	XP_005176719.1	0.0	61.33
GMOY003366		PREDICTED: C-1-tetrahydrofolate synthase, cytoplasmic isoform X3 [*Musca domestica*]	XP_005183706.1	0.0	36.28
GMOY004222	GluR-gf3: ionotropic glutamate receptor	hypothetical protein FF38_07882 [*Lucilia cuprina*]	KNC24884.1	0.0	10.41
GMOY004447		hypothetical protein FF38_11623 [*Lucilia cuprina*]	KNC30614.1	0.0	119.17
GMOY005165	croquemort	protein croquemort [*Ceratitis capitata*]	JAB88784.1	0.0	20.13
GMOY005282	knickkopf	protein Skeletor, isoforms B/C [*Ceratitis capitata*]	JAB97494.1	0.0	40.21
GMOY005361		hypothetical protein FF38_09491 [*Lucilia cuprina*]	KNC22549.1	1.00E-68	72.52
GMOY005449		hypothetical protein FF38_14542 [*Lucilia cuprina*]	KNC32042.1	3.00E-47	50.27
GMOY005537		PREDICTED: uncharacterized protein LOC106095798 isoform X1 [*Stomoxys calcitrans*]	XP_013118620.1	0.0	241.75
GMOY005573	acid sphingomyelinase	hypothetical protein FF38_09213 [*Lucilia cuprina*]	KNC33883.1	0.0	15.57
GMOY005577		calcium-binding mitochondrial carrier protein Aralar1 [*Lucilia cuprina*]	KNC20798.1	0.0	15.44
GMOY005665		PREDICTED: uncharacterized protein LOC106084900 [*Stomoxys calcitrans*]	XP_013104316.1	0.0	52.40
GMOY005720	Zinc transporter 35C	PREDICTED: zinc transporter 2-like [*Aedes albopictus*]	XP_019550545.1	2.00E-92	178.93
GMOY006369		hypothetical protein FF38_03491 [*Lucilia cuprina*]	KNC32157.1	0.0	17.61
GMOY006417	OBP20: odorant binding protein 20	putative odorant binding protein 20 [*Haematobia irritans*]	JAV15842.1	9.00E-46	37.48
GMOY006503	Beta-hexosaminidase	PREDICTED: chitooligosaccharidolytic beta-N-acetylglucosaminidase [*Musca domestica*]	XP_005182019.1	0.0	133.63
GMOY006539		PREDICTED: alpha-1,6-mannosyl-glycoprotein 2-beta-N-acetylglucosaminyltransferase isoform X3 [*Stomoxys calcitrans*]	XP_013099543.1	8.00E-126	106.67
GMOY006677	Solute:Sodium Symporter	hypothetical protein FF38_06448 [*Lucilia cuprina*]	KNC34106.1	0.0	58.69
GMOY006713		CG8192 [*Drosophila busckii*]	ALC41544.1	2.00E-90	82.52
GMOY006818		PREDICTED: lipoma HMGIC fusion partner-like 2 protein [*Stomoxys calcitrans*]	XP_013119103.1	3.00E-118	34.98
GMOY006875		PREDICTED: alkaline phosphatase [*Musca domestica*]	XP_011291521.2	0.0	109.75
GMOY006952	Major Facilitator Superfamily transporter	Organic cation transporter-like protein [*Lucilia cuprina*]	KNC25680.1	0.0	103.42
GMOY006960		PREDICTED: venom acid phosphatase Acph-1 [*Stomoxys calcitrans*]	XP_013110448.1	0.0	22.63
GMOY007021		putative inorganic phosphate cotransporter [*Lucilia cuprina*]	KNC32863.1	0.0	65.17
GMOY007046		hypothetical protein FF38_01733 [*Lucilia cuprina*]	KNC29857.1	0.0	35.81
GMOY007169		hypothetical protein FF38_08034 [*Lucilia cuprina*]	KNC22811.1	7.00E-24	150.00
GMOY007170	Na[+]-driven anion exchanger	PREDICTED: electroneutral sodium bicarbonate exchanger 1 isoform X1 [*Stomoxys calcitrans*]	XP_013100613.1	0.0	74.61
GMOY007305		PREDICTED: lysozyme [*Drosophila suzukii*]	XP_016927878.1	2.00E-57	41.88
GMOY007468		hypothetical protein FF38_04128 [*Lucilia cuprina*]	KNC22753.1	3.00E-63	114.27
GMOY007532		putative leucine-rich repeat-containing g-protein coupled receptor 5 [*Haematobia irritans*]	JAV16303.1	0.0	77.95
GMOY007691	PAK-kinase	PREDICTED: serine/threonine-protein kinase PAK 1 isoform X1 [*Stomoxys calcitrans*]	XP_013099440.1	0.0	42.00
GMOY007960		PREDICTED: endothelin-converting enzyme 1 [*Stomoxys calcitrans*]	XP_013119452.1	0.0	187.13
GMOY008133		PREDICTED: uncharacterized protein LOC101894637 [*Musca domestica*]	XP_005176834.1	0.0	11.70
GMOY008518	sugar transporter 1	hypothetical protein FF38_06053, partial [*Lucilia cuprina*]	KNC33316.1	0.0	12.53
GMOY008765	spalt major	homeotic protein spalt-major [*Lucilia cuprina*]	KNC31339.1	0.0	100.83
GMOY009021		PREDICTED: protein D3-like [*Bactrocera dorsalis*]	XP_011197362.1	2.00E-85	157.77
GMOY009202		hypothetical protein FF38_07050 [*Lucilia cuprina*]	KNC31716.1	1.00E-47	55.01
GMOY009300		hypothetical protein FF38_11715 [*Lucilia cuprina*]	KNC30663.1	0.0	13.77
GMOY009393	beta-Tubulin at 60D	tubulin beta-3 chain [*Lucilia cuprina*]	KNC22620.1	0.0	58.23
GMOY009759		hypothetical protein FF38_02940 [*Lucilia cuprina*]	KNC28936.1	0.0	100.36
GMOY009924		PREDICTED: nuclear pore complex protein Nup133 isoform X1 [*Stomoxys calcitrans*]	XP_013101194.1	0.0	13.86
GMOY010035	Mig-2-like	PREDICTED: ras-related C3 botulinum toxin substrate 1 [*Musca domestica*]	XP_005183165.1	5.00E-143	48.84
GMOY010039	optix: optix	protein Optix [*Lucilia cuprina*]	KNC34851.1	0.0	346.87
GMOY010309	AASS: lysine-ketoglutarate reductase	hypothetical protein FF38_02955 [*Lucilia cuprina*]	KNC31958.1	0.0	8.79
GMOY010450	Trissin R: trissin receptor	PREDICTED: 5-hydroxytryptamine receptor 1 [*Bactrocera oleae*]	XP_014086219.1	0.0	372.42
GMOY010478	gl16235	PREDICTED: probable salivary secreted peptide [*Stomoxys calcitrans*]	XP_013110315.1	3.00E-58	30.94
GMOY010875	epithelial membrane protein	PREDICTED: scavenger receptor class B member 1 [*Musca domestica*]	XP_005177855.1	0.0	69.64
GMOY011278	Na[+]/H[+] hydrogen antiporter	hypothetical protein FF38_02922 [*Lucilia cuprina*]	KNC22346.1	0.0	35.31
GMOY011388		PREDICTED: lactosylceramide 4-alpha-galactosyltransferase [*Stomoxys calcitrans*]	XP_013100115.1	4.00E-136	37.41
GMOY011731	yellow-h	hypothetical protein FF38_05398 [*Lucilia cuprina*]	KNC26088.1	0.0	19.11
GMOY011839		hypothetical protein [*Ceratitis capitata*]	JAB97045.1	0.0	6.89
GMOY011858		PREDICTED: mucin-17 [*Stomoxys calcitrans*]	XP_013110461.1	2.00E-156	330.58
GMOY012079		PREDICTED: aquaporin [*Rhagoletis zephyria*]	XP_017484187.1	2.00E-74	13.87
GMOY012085	lazaro	putative phosphatidate phosphatase [*Lucilia cuprina*]	KNC34968.1	5.00E-126	93.71

### Absence of symbionts affects genes which are maturation and mating-responsive

**Fig. 3:**
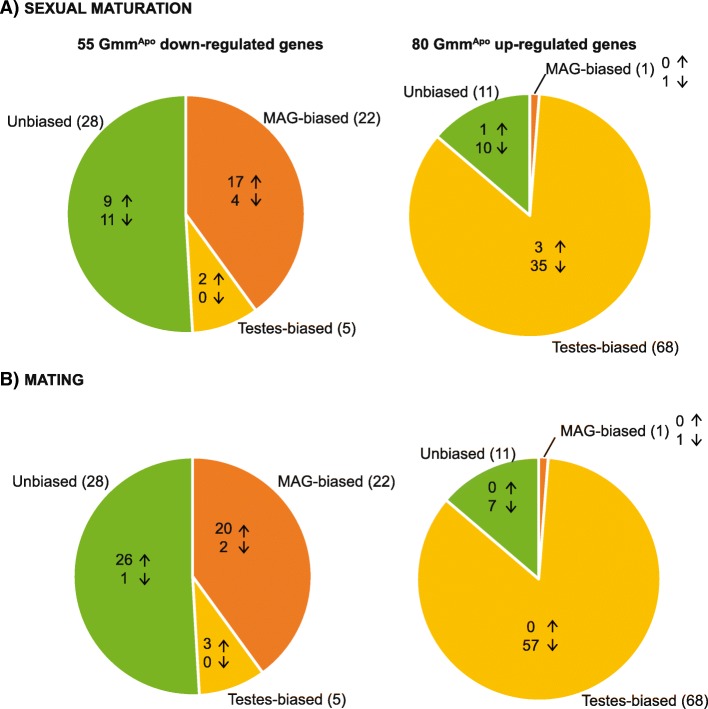
Number of genes showing a maturation and mating-responsive transcriptional profile in Gmm^WT^ male reproductive tissues. Arrows indicate up- or down-regulation in response to (**a**) sexual maturation or (**b**) mating. Genes are classified according to their biased expression profile in MAGs or testes

**Fig. 4:**
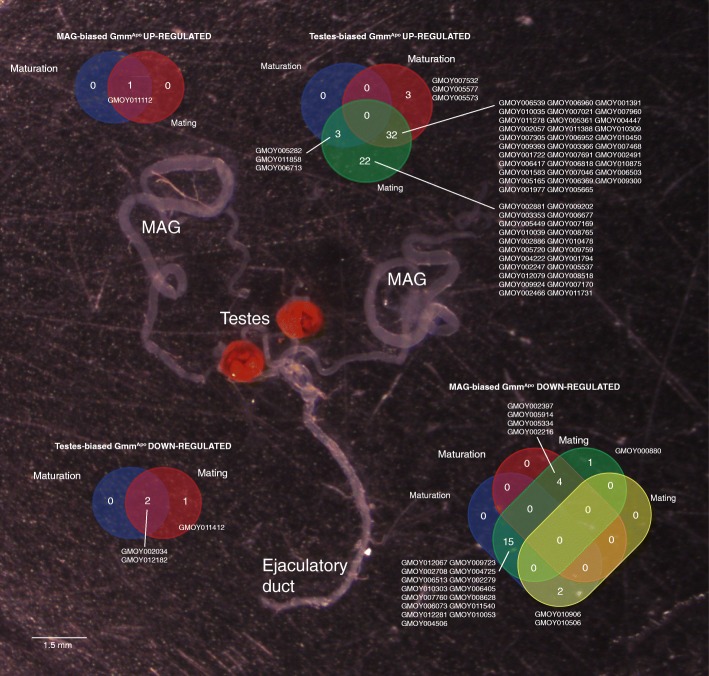
Genes showing a maturation and mating-responsive transcriptional profile in Gmm^WT^ male reproductive tissues. The IDs of MAGs- and testes-biased genes are shown in Venn diagrams illustrating the number of common sequences found between four groups, i.e. up- or down-regulated in response to maturation and up- or down-regulated in response to mating, separately for MAGs and testes. A tsetse male reproductive tract is shown. Scale bar = 1.5 mm

The genes affected by the removal of the symbionts were cross-referenced to separate libraries derived from MAGs and testes collected from Gmm^WT^ males at different sexual maturation and mating states. These libraries were derived from reproductively immature teneral flies, mature unmated and mature mated flies (6–8 h post mating) [3]. Genes were categorized as maturation- or mating-responsive using the Kal’s Z-test [30] (with FDR < 0.05) when displaying a fold change ratio > 2 (i.e. up-regulated in response to maturation/mating) and fold change ratio < 0.5 (i.e. down-regulated in response to maturation/mating). We found that, in Gmm^WT^ males, the expression of a proportion of the Gmm^Apo^ responsive genes (both down- and up-regulated) was also affected by sexual maturation (78%, *n* = 43; and 62%, *n* = 50, respectively) (Figs. 3, 4). The majority of genes down-regulated in the aposymbiotic state were, in presence of symbionts (i.e. in Gmm^WT^ males), up-regulated in response to sexual maturation (65%, *n* = 28), and were mostly MAG-biased (*n* = 17). Among these, GMOY002708, homogentisate 1,2-dioxygenase - GMOY006073, and GMOY011540 displayed a particularly high increase in response to maturation in the presence of symbionts (i.e. fold change of 6.4, 6.7, and 12.4, respectively) (Additional file 7). In contrast, the great majority of genes up-regulated in the aposymbiotic state (92%, *n* = 46) were, in the presence of symbionts (i.e. in Gmm^WT^ males), down-regulated in response to sexual maturation, and were mostly testes-biased (*n* = 35). Among these, there is the GMOY007960 gene, with yet unknown function, which showed the greatest decrease in response to maturation in normal males (45.8-fold change).

The presence of symbionts also affects expression of mating-responsive genes in both MAGs and testes (Figs. 2, 3). Indeed, in Gmm^WT^ males, the genes we found to be down-regulated by symbiont removal displayed a trend of increased transcription after mating (89%, *n* = 49), particularly in the MAGs (41%, 20). This is particularly evident in the case of the genes GMOY000880, and *kua* - GMOY005334. In contrast, the Gmm^Apo^ up-regulated genes displayed decreased transcription after mating (81%, *n* = 65), particularly in the testes (*n* = 57). Nineteen testes-biased genes displayed a particularly intense decrease in their transcription in response to mating (i.e. fold change > 50), and they include the Zinc transporter 35C - GMOY005720, GMOY007169, the Na[+]-driven anion exchanger - GMOY007170, myosin 61F - GMOY001722, and the tubulin beta 3 - GMOY009393.

### Genes which contribute to ejaculate production are down-regulated in the absence of symbionts

Among the genes displaying a significant down-regulation in Gmm^Apo^ males, we found eight transcripts that encode spermatophore proteins [3]. They included seven MAG-biased genes, namely two novel tsetse genes (i.e. GMOY004506 and GMOY007760), three genes with predicted serine protease inhibitor activity (i.e. GMOY002279, GMOY004725, and *SRPN12* - GMOY010053), one sequence with unknown function (i.e. GMOY008628), and the Angiotensin converting enzyme gene (*Ance* - GMOY009723) (Additional file 3). Interestingly, none of the Gmm^Apo^ up-regulated genes encode proteins transferred in the spermatophore.

## Discussion

*Glossina m. morsitans* aposymbiotic males have been previously shown to display a reproductive fitness similar to normal males. Indeed, they were proven able to mate with Gmm^WT^ females [12], transfer their ejaculate in the form of a spermatophore (Attardo, unpublished data), and spermatozoa were detected in the female spermathecae post-mating [12]. In addition, both larval deposition and adult progeny eclosion rates were not different from those recorded for Gmm^WT^ females mated to Gmm^WT^ males [12]. However, given the tight relationships between tsetse and its three endosymbionts, which affect multiple aspects of fly biology including metabolism and immunity [4, 31], we expect that the removal of endosymbionts may affect to some extent also male reproductive functions. To begin addressing this knowledge gap, here we generated transcriptomes from the reproductive tissues of Gmm^Apo^ and Gmm^WT^ males and identified genes differentially expressed between these two statuses, enlightening novel potentially relevant aspects of tsetse-symbionts interactions.

### Absence of symbionts depresses the activity of genes in the male accessory glands, including sequences encoding spermatophore proteins

Functions related to immunity, mating, amino acid and nucleotide metabolism, and seminal fluid proteins were directly impacted by microbiota removal in the male accessory glands. One of these functions includes chitin binding. Proteins with chitin binding activity have been associated with antimicrobial roles [32], and are found in the seminal fluid of different insects [33–38]. Two tsetse genes, which are down-regulated in the absence of symbionts, encode proteins with predicted chitin binding activity in the MAGs. One of these is GMOY000880, which bears homology to the *Drosophila* predicted chitin-binding protein CG32036 [39], and the other is GMOY002708, which carries a chitin binding Peritrophin-A domain (pfam01607: CBM_14). Our previous data on Gmm^WT^ males indicated that these two genes increase in transcription in response to mating, suggesting an immune-related activity in male reproductive function. The absence of symbionts negatively impacts the transcription of the *Glossina kua* gene (GMOY005334), which in *Drosophila* is involved in the induction of the long-term female post-mating response [40]. Whether the down-regulation of this gene impacts tsetse female post-mating behaviour remains an open question as very limited information is currently available regarding the tsetse post-mating response. In *G. morsitans*, female receptivity to remating is known to decline rapidly and almost disappear at about 48 h post-mating [41]. At the molecular level, transcription of some milk gland proteins has been found to be higher in mated and pregnant females than in unmated flies, suggesting that mating could contribute to the mechanism of milk protein activation [42].

Tyrosine detoxification is important for survival and reproduction of blood-feeding insects including kissing bugs, mosquitoes and ticks [43–45]. In tsetse, phenylalanine-tyrosine metabolism is one of the host metabolic pathways dependent on *Wigglesworthia*-derived vitamin products [4]. The *homogentisate 1,2-dioxygenase* (*hgd*) GMOY006073 is predicted to encode an enzyme mediating the conversion of homogentisate to maleylacetoacetate, a step in the catabolism of both tyrosine and phenylalanine (InterPro IPR005708). Here we found that *hgd* is down-regulated in the aposymbiotic state and this may be a consequence of a compromised vitamin metabolism due to the absence of the mutualistic symbionts in the Gmm^Apo^ line investigated. Similarly, tsetse GMOY011540 is a putative uridine phosphorylase participating in the pathways of pyrimidine ribonucleosides degradation and salvage. Its down-regulation in Gmm^Apo^ males may also result from the absence of *Wigglesworthia*, which has been proposed to assist with production of nucleotides and metabolic intermediates essential for enriched nucleotide salvage pathways [4, 46].

Only a few testes-biased genes are down-regulated in Gmm^Apo^ males. These include the neuropeptide F (NPF) GMOY012182, which, in other insects, play important roles in reproductive physiology. In *Drosophila*, NPF is involved in courtship behavior [47]. In the desert locust, *Schistocerca gregaria*, NPF is related to testis and seminal vesicle size, and may affect the biochemical composition of the seminal fluid [48]. GMOY002034 is an evolutionarily conserved SVOP synaptic vesicle transporter [49, 50]. This tsetse gene displays a sugar transporter domain. As such, it could affect the level of sugars in the ejaculate, promoting sperm motility, as proposed for *Drosophila* and as occurring in honey bees and vertebrates [40, 51].

The absence of symbionts also results in reduced transcript abundance of MAG associated genes found to encode spermatophore components in Gmm^WT^ flies. Among these, we identified genes showing serpin protease inhibitor (serpin) activity, such as GMOY002279, GMOY004725 and *SRPN12*. Serpins are the most common class of protease inhibitors in insect seminal fluid, where they play several sperm-related roles, including the mediation of sperm competition success through regulation of fertilizing capacity [52–54]. On the basis of their orthology to *Drosophila* CG16713, we could suggest the involvement of GMOY002279 and GMOY004725 in immune system responses. The presence of proteins with immune activity is not unexpected in insect seminal fluid [55–61], where they may play a protective role within the mated female reproductive tract potentially contributing to clear microbial pathogens introduced during mating [62, 63]. Indeed, CG16713, which encodes a serine protease inhibitor of the Kunitz family, is up-regulated by microbial (both bacterial, fungal, and parasitoid) infection [64–66]. Both GMOY002279 and GMOY004725 carry a Kunitz/Bovine pancreatic trypsin inhibitor domain (pfam00014) and, since in invertebrates Kunitz inhibitors are involved in a range of physiological processes including blood coagulation, fibrinolysis, inflammation and ion channel blocking, these tsetse genes may be involved in the defence against microbes in female reproductive tissues after mating [67]. Another gene potentially involved in tsetse immunity may be *SRPN12*. The *Drosophila* ortholog of this gene, Serpin 42 Da, was proposed as essential for immune defence by inhibiting several pathogenic proteolytic enzymes, due to its ability to encode for multiple protein isoforms [68, 69]. The *Larval serum protein-like 4* gene - GMOY010728, unbiased in its expression between MAGs and testes in Gmm^WT^ males, is ortholog to *pro-phenol oxidase A1* in *Drosophila*. This protein is proposed to be involved in defensive melanization responses, and is moderately up-regulated upon *Spiroplasma* infection [70, 71]. The novel tsetse proteins GMOY007760 and GMOY008628, although not displaying any conserved protein domains, carry predicted O-linked glycosylated sites. Mucin-type O-glycosylation is an evolutionary conserved protein modification and it occurs in secreted proteins involved in recognition, adhesion and cell communication events [72], suggesting that these tsetse gene products may have potential carbohydrate-mediated cell adhesion roles to support sperm function in mated females. Proteins involved in cell to cell adhesion are indeed important for fertilization in mice [73–75], and have been identified also in the seminal fluid of the tiger mosquito *Aedes albopictus* [76].

The other novel tsetse gene GMOY004506 encodes one of the six proteins that account for 50% of the spermatophore proteome [3]. Due to the abundance of its protein, GMOY004506 may play a role in the formation of the spermatophore wall. The down-regulation of the corresponding gene may have an impact on spermatophore formation/sperm transfer in Gmm^Apo^ males, potentially affecting sperm function. The *Ance* gene (GMOY009723), which is down-regulated in the absence of symbionts, may play an active role in spermatid differentiation in tsetse, as occurs in *Drosophila* [77]. This gene is proposed to be involved in the interaction between *Wolbachia* and their hosts [20, 78], and it could contribute to the Cytoplasmic Incompatibility (CI) phenotype [78]. In particular, in wRi-infected *Drosophila* cells, *Ance* was upregulated by *Wolbachia* infection [78], although this effect was not detected upon infection in *Anopheles* [79].

### Absence of symbionts activates gene expression in the testes

The up-regulated genes in Gmm^Apo^ male reproductive tissues mostly included sequences with a testes-biased transcriptional profile. These gene products are related to transport, microtubule-related structural functions, neuropeptide reception, and energy metabolism.

Several predicted transporter genes are up-regulated in the absence of symbionts. They include genes whose products are predicted to be involved in the transport of amino acids (aspartate and glutamate, in the case of the putative ortholog of *aralar1* - GMOY005577), ions and anions (GMOY005720, GMOY007169, GMOY007170), and sugars (GMOY008518). In the absence of the obligate *Wigglesworthia* symbionts, hemolymph levels of amino acids are significantly lower and carbohydrate metabolism is impaired [4]. The up-regulation of these transporters could be a response to conditions in which nutrients required for testes function are deficient. The over-expression of these genes could be an attempt by the tissue to compensate for lack of nutrients. Interestingly, the only MAG-biased gene up-regulated in Gmm^Apo^ males is a predicted organic cation transporter which, as its *Drosophila* ortholog CG6126, may affect the sugar levels in the seminal fluid as described above.

Among the testes-biased microtubule-related structural genes, we identified three up-regulated genes, *myosin IB*, *bitesize* and *tubulin β3*. *Myosin IB* (GMOY001722), as its *Drosophila* counterpart (*Myo61F*), could be involved in regulating the actin-dependent post-Golgi trafficking of cargo [80]. *Bitesize* (GMOY001977) in *Drosophila* is expressed in epithelial tissues and controls the organization of actin filaments [81, 82]. *Tubulin β3* (*betaTub60D*) up-regulation may suggest a disruption of normal axoneme assembly. In *Drosophila*, Tubulins are distributed very specifically in the testes, with *tubulin β3* being transiently expressed during mid-embryogenesis and then exclusively in cytoplasmic microtubules of somatic cells in the gonads [83, 84].

The up-regulation of genes involved in cell adhesion, such as GMOY005665 and GMOY001583, may be related to an abnormal development of germ cells. Indeed, disruption of cell-cell connections is associated with impaired sperm development and function [85, 86]. This may be the case of GMOY005665, whose best hit is the testis-specific gene CG5758, which was identified in the sperm proteome of *Drosophila* [87]. GMOY005665, as well as CG5758, carries two beta-Ig-H3/fasciclin domains (K8EG83) that are predicted to be involved in cell adhesion. The α-PS3 integrin *scab* (GMOY001583) is also involved in the regulation of adhesion, signaling, polarity and cell migration.

Potentially involved with sperm function/mobility is GMOY007960, whose best hit is the predicted endothelin-converting enzyme 1 (ECE-1) from *Stomoxys calcitrans*. In humans, ECE-1, a key enzyme in the biosynthesis of active endotelin-1 (ET-1), has been identified in prostate [88] and rat vas deferens [89]. ECE-1 transcription levels are particularly high in the Leydig cells of the human testis [90] and human seminal fluid is known to contain endothelin, which is proposed to stimulate the transport of sperm through the uterine cavity [91, 92]. In *Drosophila*, ECE-1 protein was identified in the sperm proteome [87].

## Conclusions

The results of this work revealed distinctive trends in gene differential expression in accessory glands and testes of Gmm^Apo^ males. This may be related to the fact that these two organs participate in the maintenance of complementary reproductive functions in insects. In the testes, key regulatory genes involved in spermatogenesis and sperm function tend to be conserved to guarantee the male-specific processes required for gamete production [93, 94]. However, genes in the male accessory glands are more involved in ensuring male reproductive success through the production of seminal components with effects on female post-mating response [95]. Our data suggest a role for symbionts in contributing to the proper function of these two tissues. In the MAGs, symbionts appear to affect the expression of genes related to mating, immunity and seminal fluid component synthesis. In the testes, the absence of symbionts depresses the activity of genes involved in the metabolic apparatus at the basis of male reproduction, such as sperm production, motility and function. From an applied perspective, unravelling the metabolic and functional relationships between tsetse male and its symbionts will provide basic information useful to improve Sterile Insect Technique (SIT) based programs. In particular, a deeper knowledge of the interdependencies between tsetse males and their symbionts at the levels of gene expression, protein synthesis and metabolite production/abundance could be leveraged to develop new ways to generate sterile males. Application of this knowledge to development of targeted chemical based male sterilization strategies could improve sterile male rearing, viability and competitiveness in the field.

## Methods

### Insects and sample preparation

In this work, we used male insects from the *G. morsitans* colony (Gmm^WT^) maintained at the Yale University insectary under standard laboratory conditions [96]. Aposymbiotic flies were derived as previously described [12]. Briefly, Gmm^WT^ females were fed every 48 h using an artificial feeding system on blood meals supplemented with 20 μg/ml of tetracycline and 10% (*w*/*v*) yeast extract (Becton Dickinson). Dietary supplementation with yeast extract rescues female fecundity facilitating the production of aposymbiotic offspring [12]. Blood feeding continued for the duration of their entire life span. The resulting progeny were confirmed to be aposymbiotic by PCR assays using as template genomic DNA samples from pooled male and female adult flies. DNA was extracted from the whole body of either two or eight day-old adults using the Qiagen DNeasy^R^ Blood & Tissue kit. Bacterium-specific PCR amplification was achieved using the following primer sets: *Wigglesworthia thiC* (F-5’-TGAAAACATTTGCAAAATTTG-3′; R-5’-GGTGTTACATAGCATAAAT-3′; amplicon size 365 bp); *Sodalis SscA* (F-5’ATTAAAGCGGCAGGTCATCACG-3′; R-5’-ATCGGCGGAGAACATCGGTAAG-3′; amplicon size 400 bp); *Wolbachia GroEL* (F-5’-GGTGAGCAGTTGCAAGAAGC-3′; R-5’-AGATCTTCCATCTTGATTCC-3′; amplicon size 795 bp). As a control, primers specific to tsetse *beta-1 tubulin* were used (F-5’-TCGTTGACCATGTCTGGTGT-3′; R-5’-TAGTTCTCTTCAACTTCAGCCTCTT-3′; amplicon size 650 bp). PCR reactions were performed using an MJ-Research thermocycler using the following thermal profile: 94 °C 45 s, 60 °C 45 s, 72 °C 80 s, for 35 cycles. The amplicons were visualized by electrophoresis on a 1% agarose gel. The male Gmm^Apo^ progeny flies were collected at eclosion and divided into biological replicates of 10 flies in parallel with equivalent replicates of control male flies. The flies were maintained on a normal (unmodified) blood diet. At 7 days post-eclosion, the reproductive tracts (comprising male accessory glands, testes and ejaculatory duct) were dissected from the Gmm^WT^ and Gmm^Apo^ males and stored in TRIzol® reagent (Thermo Fisher Scientific, Waltham, MA). For both normal and aposymbiotic males three biological replicates were included in the analysis [29]. Each replicate represents ten pooled male reproductive tracts.

### Library preparation and analysis

Total RNA from male reproductive tissues was extracted according to the TRIzol® reagent manufacturer’s instructions. Total RNA was treated with Ambion TURBO DNA-free DNase (Thermo Fisher Scientific, Waltham, MA). RNA quality was evaluated using an Agilent 2100 Bioanalyzer (Agilent Technologies, Santa Clara, CA). Male reproductive tract transcriptome libraries were prepared and sequenced by the Yale University Center of Genome Analysis (YCGA, New Haven, CT) using the TruSeq RNA Library Prep Kit according the standard protocols. Libraries were sequenced (75 bp single-end read) on an Illumina HiSeq 2500 machine. The RNA-seq datasets were analyzed with FastQC and trimmed in CLC Genomics Workbench (CLC Bio, Cambridge, MA) to remove ambiguous nucleotides. The trimmed datasets were mapped to *G. morsitans* transcripts (version 1.5, Vectorbase) [97]. Transcript expression levels were analyzed using CLC Genomics Workbench (CLC bio, Cambridge, MA), using RPKM as a measure of relative gene expression [98]. Genes with RPKM > 50 were considered as being significantly expressed within the library. Analysis of differential expression between the control library and treatments was performed using the EdgeR package in R (version 3.16.5) using the unique counts data derived from the CLC based mapping data. At the time the study was performed, three replicates were considered adequate to determine differential gene expression. More recent works have emphasized the inclusion of larger replicate numbers. Fortunately, the EdgeR package was designed to account for biological and technical variability through the use of empirical bayes methods to improve reliability of differential expression calls and is designed for use with low numbers of replicates [99]. Recent comparisons of RNA-seq analysis packages revealed edgeR is one of the top performing packages for controlling False Positive Rates with lower replicates primarily affecting the number of False Negatives due to the conservative nature of the model [100]. Future studies will be performed utilizing larger replicates to increase the depth of our findings. Transcripts were scored as differentially expressed if they had a *P*-value of <=0.05 and an FDR value of <=0.01. Principal component analysis (PCA) was performed using the prcomp package (version 3.3.1) in the R software suite and visualized using the Factominer (version 1.36) and factoextra (version 1.0.4) packages.

### Comparative analyses with tissue-specific RNAseq libraries and spermatophore proteome derived from Gmm^WT^ males

The identified differentially expressed transcripts were then cross-referenced to previously generated transcriptomes from normal (i.e. with full endosymbiotic fauna) males to assign: i) tissue specificity; ii) transcriptional variations in relation to sexual maturation and mating; and iii) ability to encode for spermatophore proteins. The Sequence Read Archive (SRA) numbers for the individual libraries at NCBI are SRX1254426 (testes post-mating), SRX1254425 (testes mature virgin), SRX1254417 (testes teneral), SRX1254054 (MAG post mating), SRX1251081 (MAG mature virgin), SRX1251080 (MAG teneral). Sex specificity was based on normalized gene expression calculated as the Fragments Per Kilobase of Exon per Million reads mapped (FPKM), analysed using the Baggerley’s test [101] and filtered with the False Discovery Rate (FDR) *P*-value correction of 0.0001, in accordance to our previous work [3]. To assign tissue-specificity, we combined the values for the three replicates corresponding to the testes and the MAG libraries we previously produced (i.e. teneral, 3 days old mature virgin, and mated males), respectively, and derived for each transcript an average expression value for each of the two tissues as we described previously [3]. Briefly, in all comparisons, we considered one gene to be tissue-biased when fold change in one tissue was at least 5-fold higher in expression than in the other. One gene was considered to be tissue-specifically expressed when fold change in one tissue was at least 5 folds higher in expression than in the other and the number of unique reads in the other tissue was less than 50.

To assign transcriptional variations in relation to sexual maturation and mating, we individually used the six different libraries derived from MAGs and testes from teneral, mature virgin and mated males, respectively, and derived fold change variations as previously described [3]. Briefly, normalized gene expression calculated as FPKM were analysed using the Kal’s Z-test [30] with FDR P-value correction < 0.05. Fold change ratio between the immature males (MAGs or testes tissues) and mature virgin (MAGs or testes tissues) normalized expression means were derived and we defined a gene as maturation-biased when displaying a fold change ratio > 2 (i.e. up-regulated in response to maturation) and fold change ratio < 0.5 (i.e. down-regulated in response to maturation). The intermediate ratios were considered unbiased. The fold change ratios between mature virgin (MAGs or testes) and mated (MAGs or testes) normalized expression means were similarly analyzed and allowed to define mating-responsive genes. The intersections among the four groups (i.e. up- or down-regulated in response to maturation and up- or down-regulated in response to mating) of MAG- or testes-biased genes were visualized by venn diagrams [102].

In addition, we cross-referenced the dataset of 135 symbiont-responsive genes with genes corresponding to the identified 287 spermatophore proteins and determined whether, in Gmm^WT^ individuals, they were encoding for transferred seminal fluid proteins [3]. O-glycosylation sites in spermatophore proteins were predicted with the program NetOGlyc [103].

## Additional files

Additional file 1: The effect of tetracycline treatment on the maternal transmission of tsetse endosymbionts. PCR assays on genomic DNA showing that Gmm^WT^ flies were positive for *Wigglesworthia* (Wig Thic), *Sodalis* (Sod SsaC) and *Wolbachia* (Wol GroEL), whereas offspring resulting from tetracycline-treated flies lack all three symbionts. The bottom panel shows the amplification of tsetse tubulin beta-1 gene on all samples. A and B lanes represent two PCR replicates performed on pooled genomic DNA from ten males and ten females extracted either at 2 and 8 days post-emergence. (PDF 1070 kb)

Additional file 2: Principal Component Analysis of gene expression in Gmm^WT^ and Gmm^Apo^ male reproductive tissues. (PDF 98 kb)

Additional file 3: Excel spreadsheet containing the descriptions of the differentially expressed genes in the reproductive tract tissues between Gmm^WT^ and Gmm^Apo^ males. (XLSX 67 kb)

Additional file 4: Gene Ontology analysis of genes down-regulated in Gmm^Apo^ male reproductive tissues. The number of genes associated with corresponding Gene Ontology terms (Biological Process, Molecular Function, and Cellular Component Level III) is shown. (PDF 784 kb)

Additional file 5: Gene Ontology analysis of genes up-regulated in Gmm^Apo^ male reproductive tissues. The number of genes associated with corresponding Gene Ontology terms (Biological Process, Molecular Function, and Cellular Component Level III) is shown. (PDF 778 kb)

Additional file 6: Excel spreadsheet showing the Gene Ontology terms associated to genes down- and up-regulated in Gmm^Apo^ male reproductive tissues. (XLSX 54 kb)

Additional file 7: Changes in transcript abundance in response to maturation and mating in the presence of symbionts of genes down- and up-regulated in Gmm^Apo^ male reproductive tissues. Fold change difference in expression is separately shown for MAG- and testes-biased genes for A) Gmm^Apo^ down-regulated, and B) Gmm^Apo^ up-regulated genes. (PDF 893 kb)

## Abbreviations

CI: Cytoplasmic Incompatibility
FDR: False Discovery Rate
*Gmm*^Apo^: Aposymbiotic *G. morsitans*
Gmm^WT^: Wild-type *G. morsitans*
GO: Gene Ontology
MAGs: Male Accessory Glands
PCA: Principal Component Analysis
SIT: Sterile Insect Technique
