# Supplementary figures and images for: Symbiotic microbes affect the expression of male reproductive genes in Glossina m. morsitans
Source: BMC Microbiol. 2018 Nov 23;18(Suppl 1):169. doi: 10.1186/s12866-018-1289-2 (PMC6251095; doi:10.1186/s12866-018-1289-2)

# Tetracycline treated

Day 2

Day 8

WT

A

B

WT

A

B

Wig  
ThiC

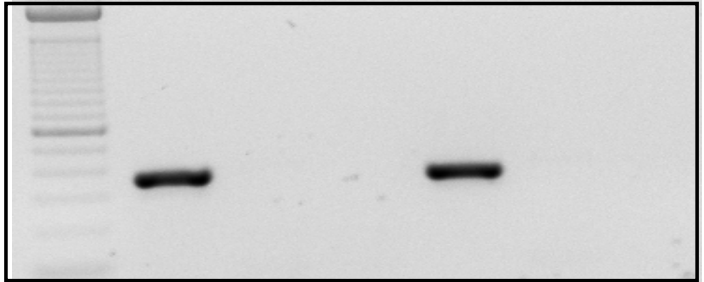

Sod  
SsaC

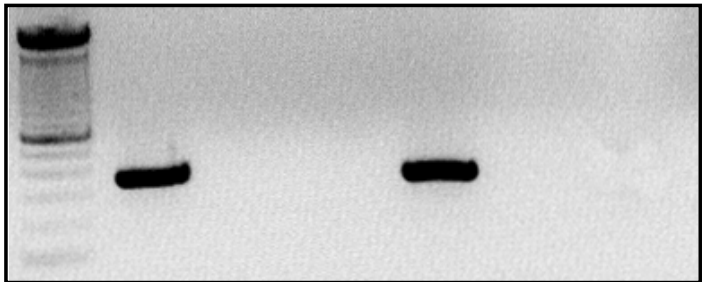

Wol  
GroEL

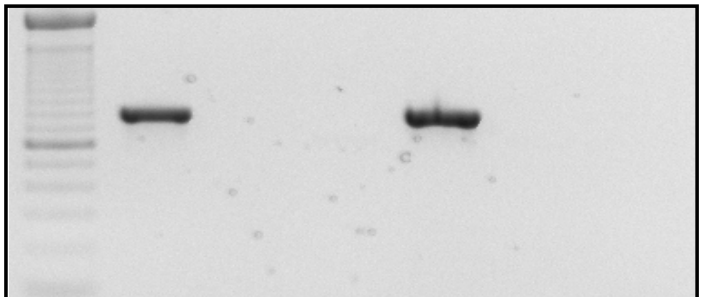

Tse  
Tub

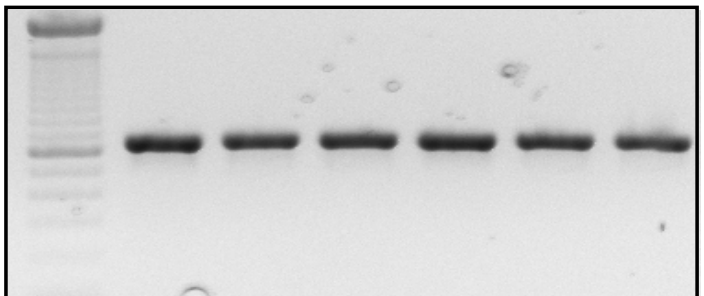

Supplement: Supplementary file 1 — The effect of tetracycline treatment on the maternal transmission of tsetse endosymbionts. PCR assays on genomic DNA showing that GmmWT flies were positive for Wigglesworthia (Wig Thic), Sodalis (Sod SsaC) and Wolbachia (Wol GroEL), whereas offspring resulting from tetracycline-treated flies lack all three symbionts. The bottom panel shows the amplification of tsetse tubulin beta-1 gene on all samples. A and B lanes represent two PCR replicates performed on pooled genomic DNA from ten males and ten females extracted either at 2 and 8 days post-emergence. (PDF 1070 kb) [file 12866_2018_1289_MOESM1_ESM.pdf]

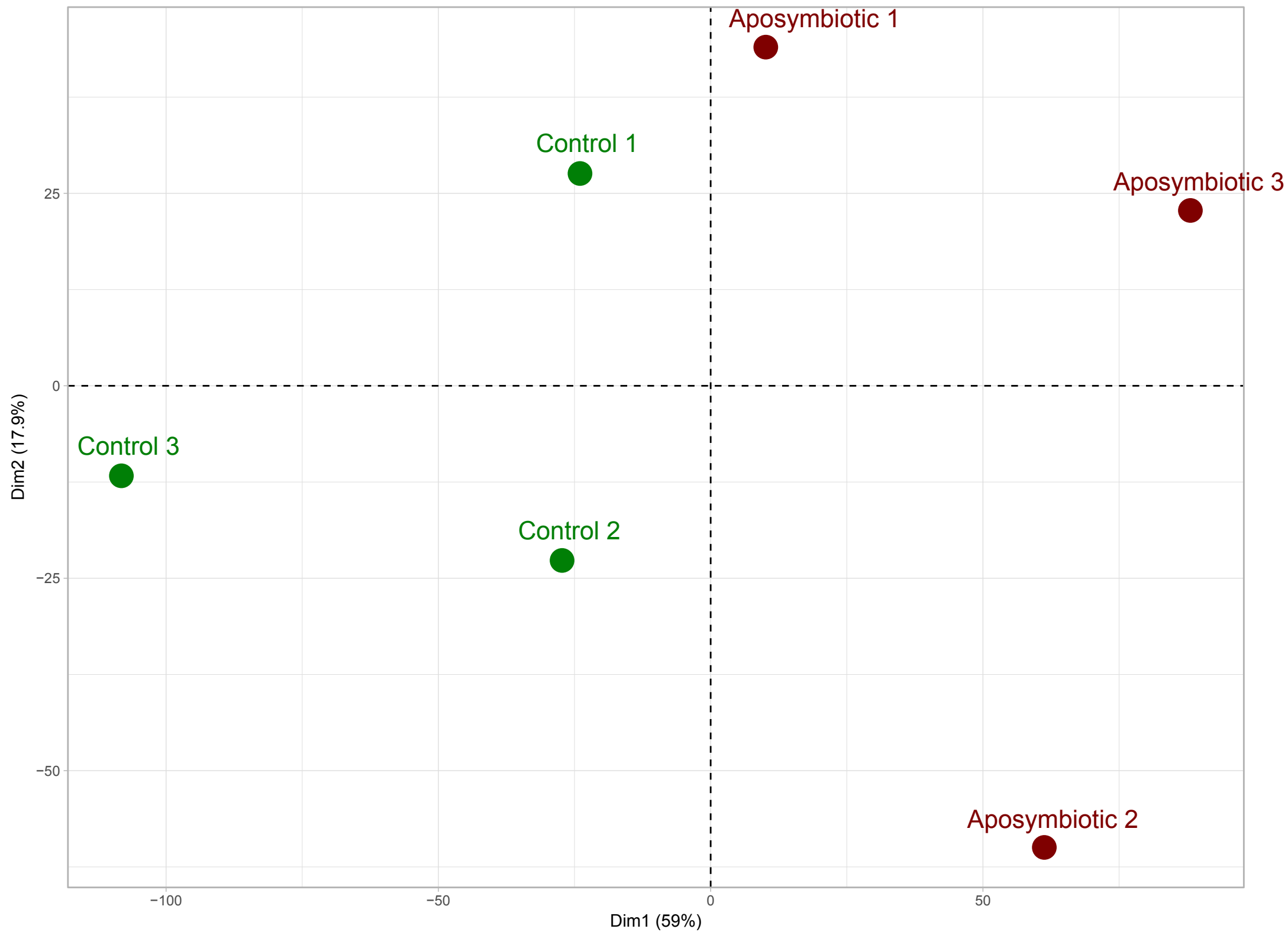

Supplement: Supplementary file 2 — Principal Component Analysis of gene expression in GmmWT and GmmApo male reproductive tissues. (PDF 98 kb) [file 12866_2018_1289_MOESM2_ESM.pdf]

## Biological Process Terms

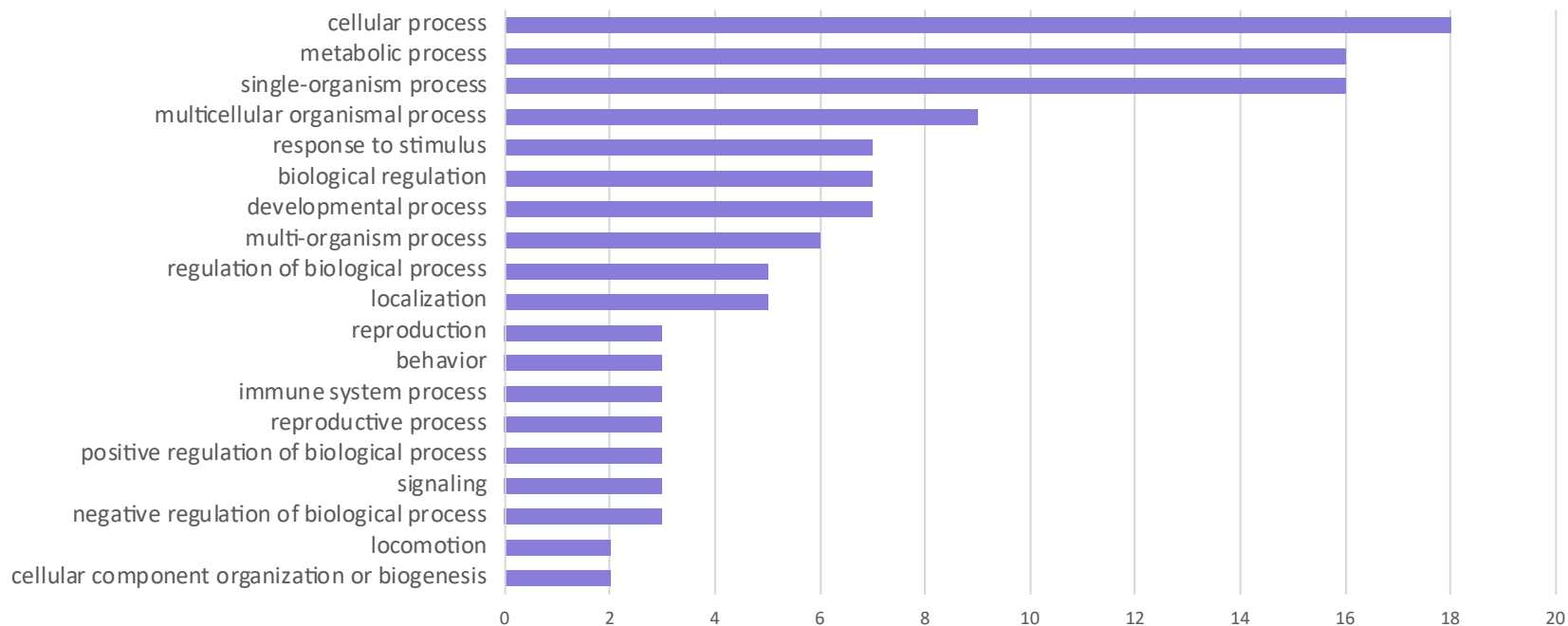

## Molecular Function Terms

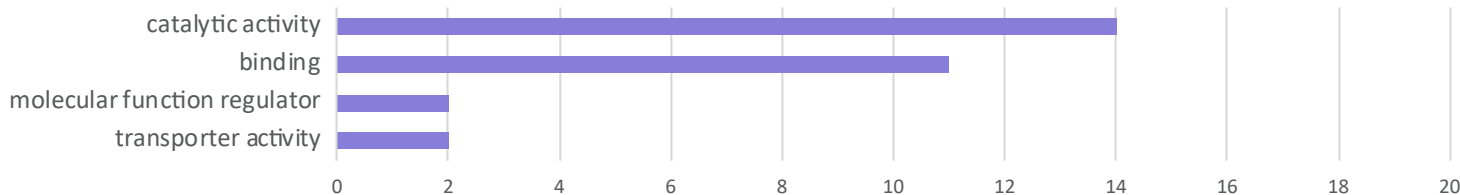

## Cellular Component Terms

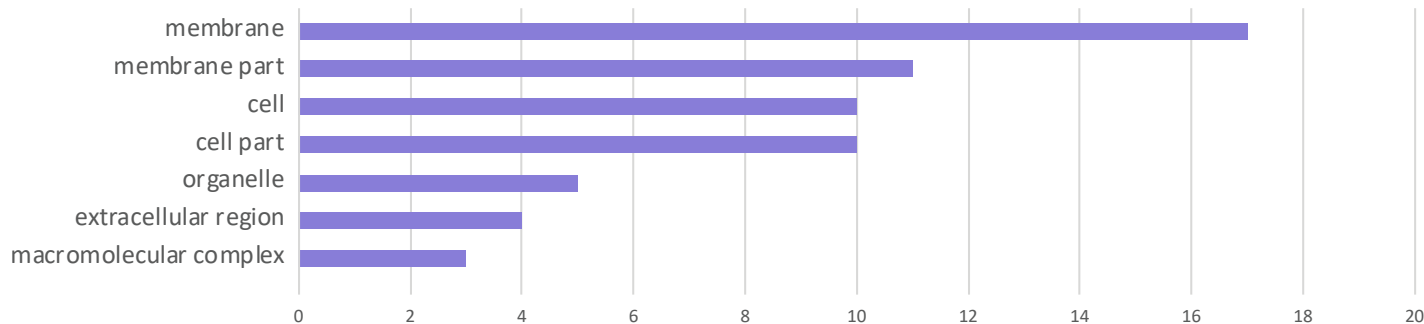

Supplement: Supplementary file 4 — Gene Ontology analysis of genes down-regulated in GmmApo male reproductive tissues. The number of genes associated with corresponding Gene Ontology terms (Biological Process, Molecular Function, and Cellular Component Level III) is shown. (PDF 784 kb) [file 12866_2018_1289_MOESM4_ESM.pdf]

# Biological Process Terms

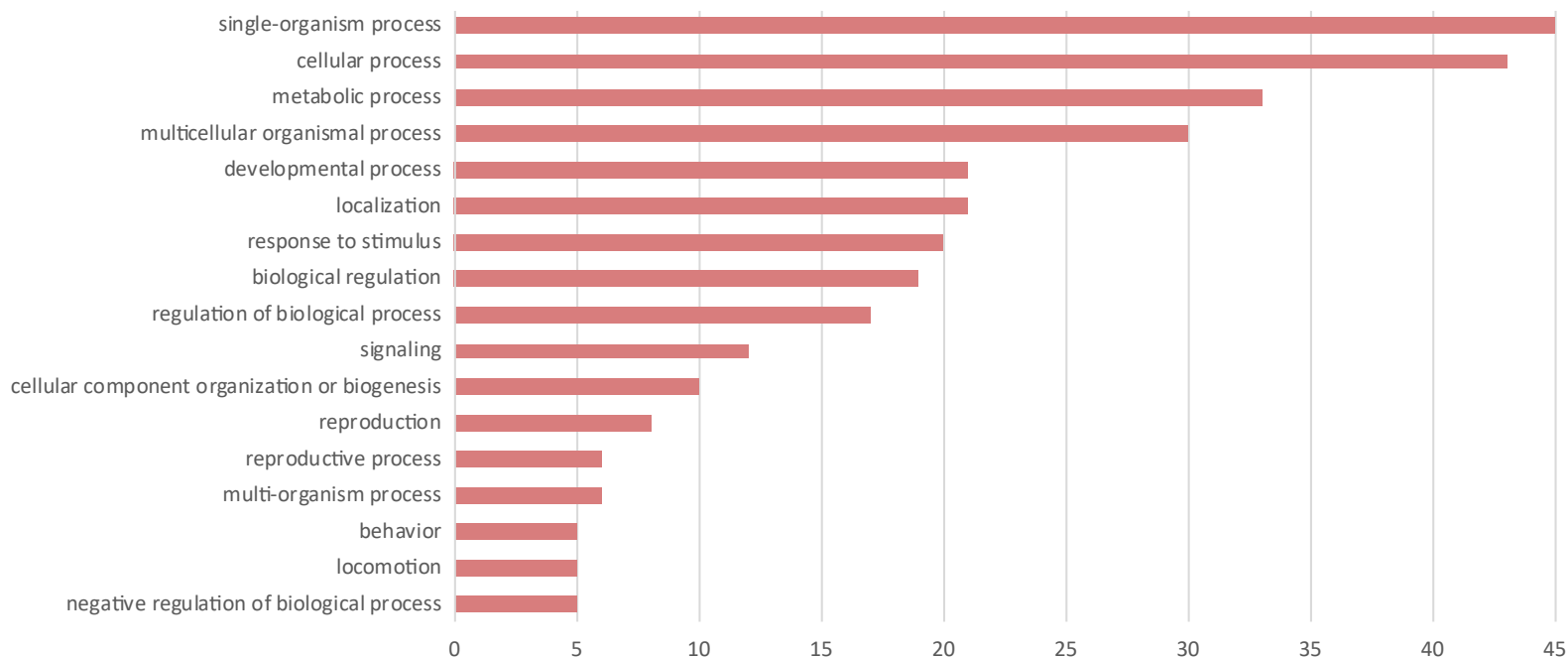

# Molecular Function Terms

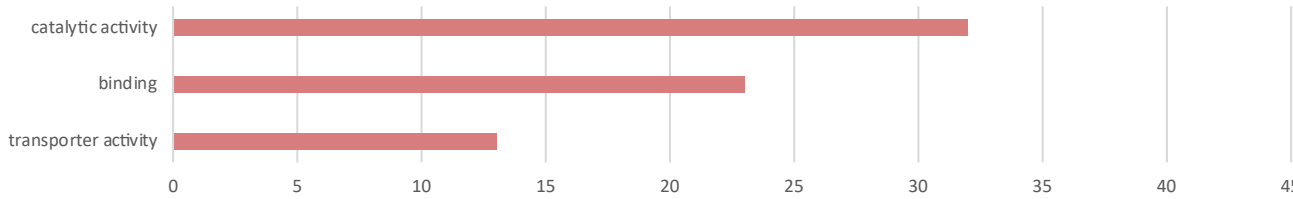

# Cellular Component Terms

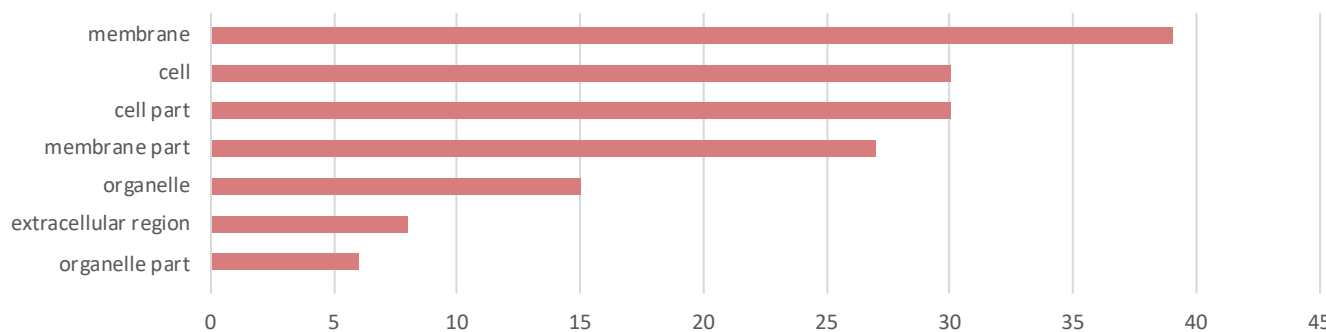

Supplement: Supplementary file 5 — Gene Ontology analysis of genes up-regulated in GmmApo male reproductive tissues. The number of genes associated with corresponding Gene Ontology terms (Biological Process, Molecular Function, and Cellular Component Level III) is shown. (PDF 778 kb) [file 12866_2018_1289_MOESM5_ESM.pdf]

A) 55 Gmm<sup>Apo</sup> down-regulated genes

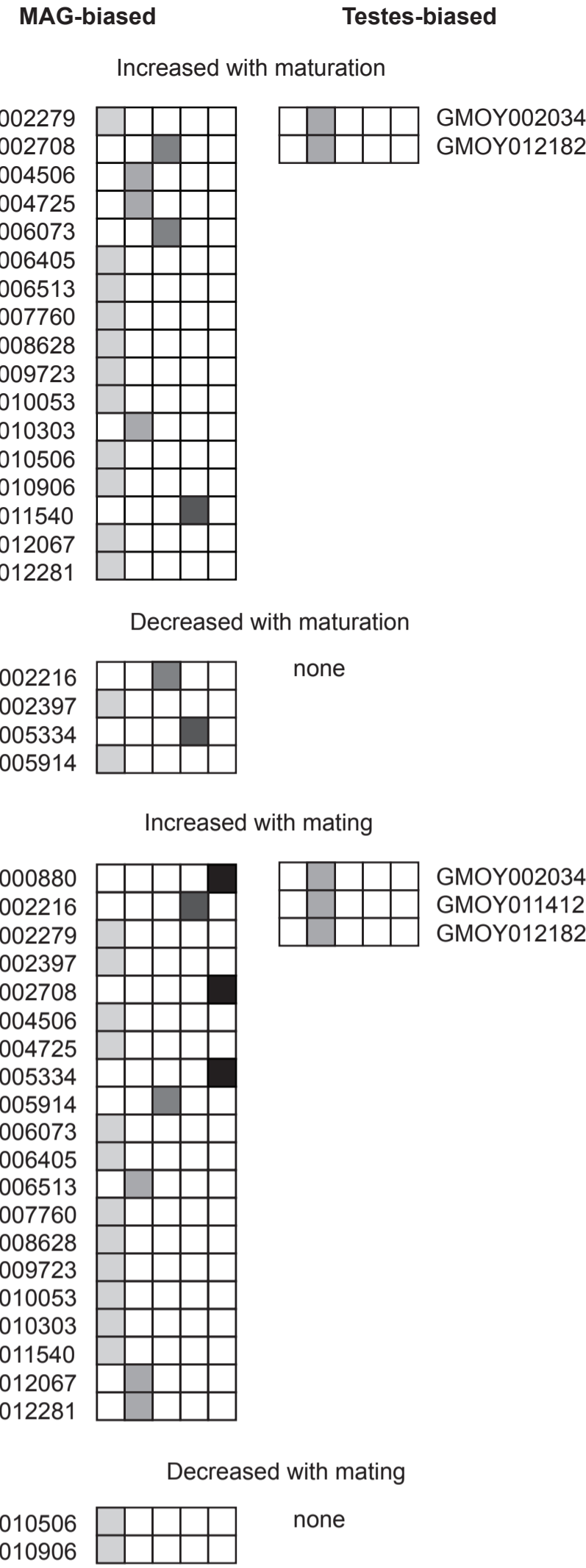

B) 80 Gmm<sup>Apo</sup> up-regulated genes

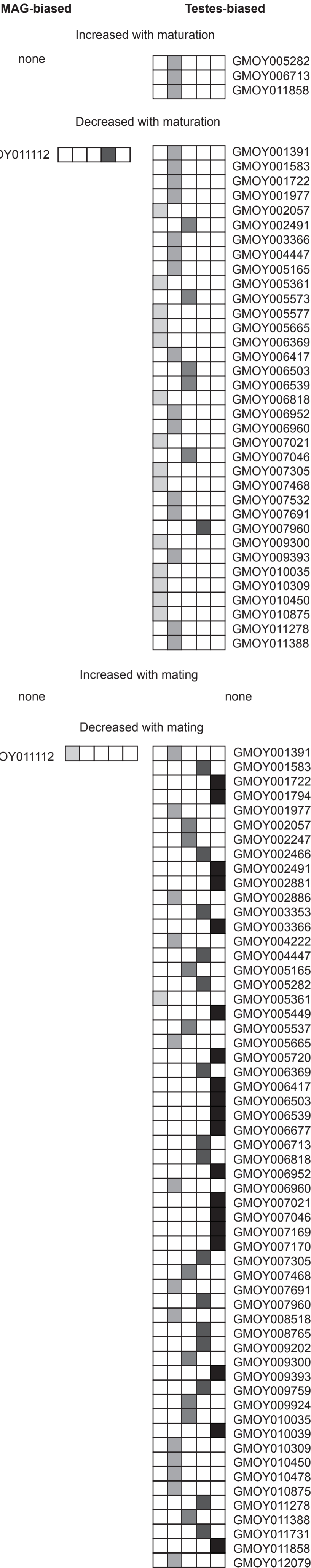

Supplement: Supplementary file 7 — Changes in transcript abundance in response to maturation and mating in the presence of symbionts of genes down- and up-regulated in GmmApo male reproductive tissues. Fold change difference in expression is separately shown for MAG- and testes-biased genes for A) GmmApo down-regulated, and B) GmmApo up-regulated genes. (PDF 893 kb) [file 12866_2018_1289_MOESM7_ESM.pdf]
